# Optimizing Our Patients’ Entropy Production as Therapy? Hypotheses Originating from the Physics of Physiology

**DOI:** 10.3390/e22101095

**Published:** 2020-09-29

**Authors:** Andrew J. E. Seely

**Affiliations:** 1 Faculty of Medicine, University of Ottawa, Ottawa, ON K1H 8M5, Canada; aseely@ohri.ca; 2 Ottawa Hospital Research Institute, University of Ottawa, ON K1Y 4E9, Canada; 3 Thoracic Surgery and Critical Care Medicine, University of Ottawa, ON K1H 8L6, Canada

**Keywords:** maximum entropy production principle, fractal structures, complex non-equilibrium systems, monitoring of scale-invariant variation, thermodynamics

## Abstract

Understanding how nature drives entropy production offers novel insights regarding patient care. Whilst energy is always preserved and energy gradients irreversibly dissipate (thus producing entropy), increasing evidence suggests that they do so in the most optimal means possible. For living complex non-equilibrium systems to create a healthy internal emergent order, they must continuously produce entropy over time. The Maximum Entropy Production Principle (MEPP) highlights nature’s drive for non-equilibrium systems to augment their entropy production if possible. This physical drive is hypothesized to be responsible for the spontaneous formation of fractal structures in space (e.g., multi-scale self-similar tree-like vascular structures that optimize delivery to and clearance from an organ system) and time (e.g., complex heart and respiratory rate variability); both are ubiquitous and essential for physiology and health. Second, human entropy production, measured by heat production divided by temperature, is hypothesized to relate to both metabolism and consciousness, dissipating oxidative energy gradients and reducing information into meaning and memory, respectively. Third, both MEPP and natural selection are hypothesized to drive enhanced functioning and adaptability, selecting states with robust basilar entropy production, as well as the capacity to enhance entropy production in response to exercise, heat stress, and illness. Finally, a targeted focus on optimizing our patients’ entropy production has the potential to improve health and clinical outcomes. With the implications of developing a novel understanding of health, illness, and treatment strategies, further exploration of this uncharted ground will offer value.

## 1. Introduction

Physicians are fundamentally physicists at heart, seeking to understand *why* their patients are ill, so that they can improve the care they provide. Given that our patients (and indeed all of life) are governed by the physics of non-equilibrium thermodynamics (i.e., systems of flow), and due to novel developments in the understanding of non-equilibrium systems, there may be value in now bringing this science to the bedside. As we know, our ability to continuously burn oxygen into carbon dioxide is essential for our health (i.e., we begin to die without oxygen metabolism in minutes); what we do not generally appreciate is how this is central to the process of entropy production. In addition, as we shall see, entropy production is spontaneous and pursues maximal states in complex non-equilibrium systems, helping us to understand the origins of healthy physiological structures. Entropy production may also be described in terms of energy dissipation and information reduction. Understanding the clinical importance of entropy production requires exploring the thermodynamic physics of physiology.

In this article, novel concepts are reviewed and discussed at a high level to try to enable a broad multidisciplinary readership, with the ultimate aim of leading to enhancing our understanding of health and illness. This is an exploratory paper lacking in quantitative rigor, respectful of the complex subject matter and the scientists who perform more in depth study of the topics discussed. The ideas presented are principally intended to link previously unexplored connections and stimulate novel questions, experiments, and discussions, rather than offer confirmation or proof. However, as I hope you will agree after reading this work, there is compelling evidence to suggest that nature’s pursuit of optimal entropy production may be a critical partner to natural selection in our evolution; explain why ordered structures ubiquitously and spontaneously form within biology and nature; and help uncover the origins and self-organization of metabolism, healing, and consciousness. Experiments evaluating or disproving these hypotheses are suggested. The aim is to stimulate critical appraisal and an improved understanding of the physics of physiology, in order to better care for our patients.

### 1.1. Introduction to Entropy

It is essential to begin with a brief review of the scientific concept of entropy. The First Law of Thermodynamics informs us that energy is always conserved, and the Second Law teaches us that energy gradients always disperse. In other words, while the amount is conserved, energy gradients spontaneously dissipate, reducing energy’s ability to perform work, and the loss of energy gradients is measured as increasing entropy [1]. Within any isolated system, which is either closed (i.e., no interaction of energy or matter with its environment) or includes the system and its environment if there is interaction, the Second Law states that the change in entropy of an isolated system is universally positive (i.e., greater than or equal to zero). This is a probabilistic law, as Ludwig Boltzman (1844–1906) developed the atomistic theory of matter, linking entropy to a probability-based arrangement of matter, where the Second Law highlights nature’s drive to pursue states with more accessible microstates (i.e., more microscopic configurations), equivalent to greater disorder and randomness, producing a homogenous high-probability soup. However, entropy should not be considered a “measure of disorder” [2,3], and a more accurate and helpful way of envisioning entropy is through its proclivity to eliminate energy gradients. If energy gradients exist, entropy will be spontaneously generated as gradients spontaneously and irreversibly degrade (unless new energy is added to maintain the gradient), as “nature abhors a gradient” [4]. As increases in entropy in terms of matter and energy are irreversible, the Second Law provides directionality to time, always moving forward with an increasing entropy of all isolated systems, including the universe. If this were the end of the story, we would observe that nature would simply degrade into maximally dispersed energy gradients (i.e., an orderless soup). However, in striking contrast, nature and life are characterized by the continuous creation of remarkable ordered complex systems that are far from thermodynamic equilibrium. As such, there has been an increased focus on non-equilibrium thermodynamics, with a particular focus on physics [5], chemistry [6], and other disciplines [7]. In fact, it is precisely by continuously producing entropy (i.e., degrading energy gradients) that life spontaneously creates its internal order.

### 1.2. Entropy Production and Life

Why is entropy production important to life? First, it is important to note that while it is possible in theory to conceptualize the entropy content of biological organisms, in practice, it is impossible to measure it. Instead, it is both possible and useful to measure the *entropy production* of a living biological system [8]. If we consider humans as living complex systems with dermal boundaries, it is consistently observed that we must continuously import free energy (i.e., O_2_, glucose, H_2_O, and food) and continuously export waste (i.e., CO_2_, urine, and stools), until we stop doing so, signifying the end of our life. As famously highlighted by Edwin Schrödinger (1887–1961) in 1944, life both derives “order from order” from generation to generation (i.e., he postulated the necessity of a genetic code), and “order from disorder” (i.e., internal “order” must be accompanied by a greater release of “disorder” to the environment) [9]. What he realized was that as humans continuously interact with their environment, the Second Law states that the entropy they produce must be greater than the negative entropy required for maintaining their ordered internal structure; or simply, humans must produce entropy to survive.

Entropy production occurs on every scale within the ‘system of systems’ that make up human organisms. Internally, gradients driving reactions, cycles, and flows occurring within and between organelles, cells, and organs indicate local entropy production, reflecting “internal irreversibilities” participating in metabolism, where the majority are released as heat; “Apart from thermodynamics governing internal biological processes, there is a perpetual outflow of energy in the form of heat loss, Q and hence disposal of entropy generated within the whole body in the form of heat to the environment” [10]. Heat is defined as a process of energy transfer, which we must continue to shed to maintain our wellbeing [11,12]. For a human organism as a whole, as is the case for any thermodynamic body, entropy production is equal to heat transfer (Q) divided by temperature (T), and entropy units are thus Joules/Kelvin.

How should we measure human entropy production? Ichiro Aoki calculates human entropy production through irreversible energy and mass flows, such as infrared radiation, conduction, convection, respiration, and evaporation [8]. By studying entropy production in multiple living systems, Aoki has noted that all living creatures go through a process of initial rising entropy production and then stability, followed by a period of slow decline, and finally a period of deterioration to zero, synonymous with death [8,13]. While a quantitative definitive study on how oxygen metabolism and heat production change along with entropy production in humans in states of health, illness, and exercise has not yet been performed, it appears likely that oxygen metabolism, heat production, and entropy production are closely related. In chemical reactions where the temperature is fixed, the amount of heat produced per unit time closely relates to entropy production [11], and with individual cells, entropy production to the environment external to the cell is largely comprised of heat [12]. As oxygen metabolism increases with exercise, heat production concomitantly increases, depending on the metabolic efficiency [14]. Aoki’s graphical representation of the recurring pattern of entropy production over a lifespan, namely a sigmoidal-shaped initial rise in entropy production, followed by a gently falling plateau period and loss during aging leading to death, is precisely similar to the pattern of VO_2_max levels observed during growth, middle age, illness, and dying [15,16,17]. Studies on the total entropy production of a human organism, modeled as a sum of the entropy generation of internal organs throughout a lifespan, have been performed [10]. Therefore, human entropy production, related to heat production divided by temperature, is closely related to oxygen metabolism, which is vital to human life. However, prior to exploring clinical implications, the further development of non-equilibrium thermodynamics will offer new and important insights.

## 2. The Maximum Entropy Production Principle (MEPP)

Derived from atmospheric and then multidisciplinary science, increasing evidence suggests that complex non-equilibrium systems, characterized by continuous energy dissipation (and thus entropy production), will adopt stable steady states involving maximal entropy production (given system constraints), such that the energy dissipation and entropy production will not only occur spontaneously, but will do so in the most efficient way possible. This Maximum Entropy Production Principle (MEPP) has been stated by Kleidon as follows: *“Non-equilibrium systems organize in a steady state such that the rate of entropy production is maximized”* [18]. A recurring example of a non-equilibrium system that maximizes entropy production would include a whirlpool, tornado, or hurricane, all of which dissipate energy gradients.

MEPP is distinct from, yet closely related to, broader statements referred to as the Law of Maximum Entropy Production (i.e., a system will select a path or assembly of paths out of available paths that minimizes the potential or maximizes the entropy at the fastest rate given the constraints) [19,20], or the Fourth Law of Thermodynamics (i.e., systems increase entropy at the maximum rate available to them [21], or more recently, evolution (of a system) occurs in the direction of the steepest entropy ascent compatible with constraints) [22]. In other words, not only will water flow down a hill dissipating gravitational potential energy (Second Law), it will do so in the most efficient way possible (Fourth Law).

In addition, others have characterized closely related concepts in different terms. Began has highlighted the concept of maximizing flow (e.g., a flow system must evolve such that it provides greater access to its currents) [23], and Chiasson has highlighted the concept of maximizing the energy rate density (i.e., the free energy flow through a system) [24]. While important differences exist, what all of these concepts similarly highlight is the concept of a selection process whereby a system will spontaneously select a state or path.

Returning to human physiology, we demonstrate emergent systemic qualities from immune-inflammatory function to psychological identity, we maintain a non-equilibrium interaction with our environment, we demonstrate the capacity to respond to stimuli, and we process information into meaning and memory. As such, we are *complex dissipative adaptive cognitive systems*, and thus, it is MEPP that is relevant for us. While the range of applicability of MEPP continues to be investigated [25,26,27,28,29,30], its application is supported in all complex non-equilibrium systems which display “emergence”, that is, systemic features that arise de novo from the integrity of the whole [3,31]. MEPP has demonstrated effectiveness in solutions of broad ranging multidisciplinary problems [3,11,32,33,34], and here, our focus is developing an improved understanding of our patients and their care.

### MEPP and the Origin of Metabolism and Physiological Ordered Structures

The first link to physiology lies with the origins of oxygen metabolism. As discussed, MEPP states that a complex non-equilibrium system will adopt an internal state of greater complexity (i.e., complex order) if the state is associated with a greater energy flow, which enables greater energy gradient dissipation and thus greater entropy production; ordered structures form spontaneously to enhance energy flows. Examples are replete in nature, from Bénard cells (i.e., the spontaneous appearance of currents in liquid layers to enhance energy dissipation) and the spontaneous formation of a whirlpool that enhances the flow down the drain of a bathtub, to tornados, hurricanes, and more. This physical force selects paths of system change to select states that enhance energy flow. Metabolism, which is the ‘burning’ of oxygen (along with organic compounds such as glucose) to carbon dioxide (and water), is the principal means through which aerobic cells and organisms produce heat and entropy. Indeed, a reduction of oxygen (i.e., electron gain) provides “close to the largest possible transfer of energy for each electron transfer reaction”, and this steep thermodynamic energy gradient of oxygen is believed to be critical to the development of multicellular complexity [35]. Based on Martyushev and Seleznev’s overview of MEPP [11], others have demonstrated that metabolic networks evolve to a state of maximum entropy production [36,37]. Furthermore, if a cooperative collective of multiple cells is capable of greater entropy production, then nature’s physical drive to enhance entropy production may help drive the spontaneous leap from single to multi-cellular organisms. Analogously, the growth from towns to cities has been enabled by the capacity to allow for a greater energy flow into a city and clearance of waste from it [38]. Given the relationship between temperature and metabolism (discussed below), MEPP is hypothesized to help ‘select’ the average human temperature of 37.5 °C if it optimizes the quotient of heat production divided by the temperature. Therefore, according to MEPP, the origin of oxygen burning metabolism arose from the dynamics of living systems adjusting themselves to maximize their entropy production [39].

In addition to having a metabolic function, MEPP may be the reason why characteristic physiological structures spontaneously form over time and space. Specifically, the ubiquitous presence of fractal structures (i.e., tree-like structures that demonstrate multi-scale self-similarity) are hypothesized to have originated because they optimize entropy production [40]. Everywhere in nature (including anatomy and physiology), fractal physical structures spontaneously form, demonstrating bounded multi-scale self-similarity that displays similar characteristic patterns (e.g., branching and waves) over multiple scales of magnitude within the scale of the system. Spatially, fractal structures (e.g., trees, mountains, coastlines, river deltas, lightning, tornados, and whirlpools) appear ubiquitously, demonstrating bounded multi-scale self-similarity. Anatomically, alterations in these structures are associated with systemic change (i.e., illness) in patients (e.g., an altered tracheobronchial tree structure in asthma [41,42], altered vasculature in stroke [43,44], and altered CNS fractal dimensions in brain pathology [45]). Temporally, nature is also replete with complex time-series, which display power-law dynamics, again with bounded multi-scale self-similarity. These characteristics are found with heart rate variability (HRV) and respiratory rate variability (RRV), whose complexity characteristics are preserved in health and reduced with illness, stress, and ageing. There are numerous techniques that can be applied to measure variability [46,47,48,49], which have been used to demonstrated that altered HRV is associated with renal failure [50,51], heart failure [52,53], angina [54], diabetes [55], hypertension [56], myocardial infarction [57,58], coronary artery disease [59], infection [60,61,62], and organ failure [63], and altered RRV is associated with respiratory illness [64] and extubation outcomes [65,66]. By applying MEPP to understand the origin of fractal heart and respiratory rate dynamics, we previously hypothesized that the fractal structure of HRV and RRV develops as a self-organizing emergent event, spontaneously occurring and continuously enabling the system to optimize its entropy production [40]. Therefore, nature’s drive to optimize entropy production is hypothesized to explain why fractal structures are ubiquitous and spontaneously self-organizing in nature. For example, the fractal geometry of coastlines serves as an attractor, with coastlines universally and naturally converging to a fractal shape, as irregular coastlines help dampen sea waves [67]. All of this applies to the origin of complex structures observed in physical (i.e., mountain ranges and coastlines) and geological (i.e., Richter’s Law) systems, as well as biological systems in space (e.g., fractal vascular trees) and time (e.g., HRV and RRV). Therefore, the physical force of MEPP may play a central and indeed underappreciated role in the origin of structures and processes within non-equilibrium systems; however, biological systems have also been driven by a separate and complementary force, namely evolution.

## 3. Interaction between MEPP and Evolution

Evolution and entropy are critical to understanding biology; evolution has guided the selection of genetic mutations that offer survival advantages, while the inexorable drive towards increasing entropy leads to decay, breakdown, and death. However, the MEPP offers a critical additional piece of the puzzle. If the drive to dissipate energy gradients leads to complex dissipative structures that are a natural physical phenomenon ubiquitous in nature from whirlpools and hurricanes to trees and cities, then what impact does evolution separately have in the biological world, where selection based on survival for the purposes of reproduction has played a central role over millennia? Skene has evaluated evolution from a thermodynamic perspective, including the MEPP, finding that MEPP provides a complementary directionality to Darwinian natural selection [68]. One of the controversies in evolution has been the question of why evolution is associated with an increasing complexity from protozoa to primates, as there is no a priori reason to expect evolution to select for complexity; MEPP offers an explanation for this. As Zotin and Lamprecht have demonstrated, the oxygen consumption rate per unit mass has steadily increased over the course of evolution [69]. Martyushev highlights that this is consistent with MEPP, stating that “According to the generalized formulation of MEPP: at each hierarchical level, the system will choose the state with the maximum entropy production density (specific heat production)”, thus “providing a directionality or progressiveness to evolution” complementary to selection for survival [3]. MEPP is consistent with Lotka’s original observations that “evolution proceeds in such direction as to make total energy flux through the system a maximum compatible with the constraints” [70]. In addition, going beyond the animate world, Chiasson has observed a remarkable pattern of increasing energy flow normalized by system size (measure in ergs per sec per gram) universally in inanimate and animate non-equilibrium systems since the origin of the universe; in fact, the energy flow density correlates so closely with system complexity that the author suggests it represents a reasonable measure of complexity itself [38]. There has been a progressive increase in the free energy flow density (and thus entropy production) from stars to planets, plants, animals, brains, and cities [24]. Regardless of this, these observations suggest that nature’s drive to augment energy dissipation or entropy production is vitally important, along with natural selection, in understanding the origins and evolution (i.e., rise) of biological complexity.

### Impact of Evolution and Maximum Entropy Production on Human Health

Returning to the clinical realm of physiology, the question remains of how these two selection principles, namely MEPP and evolution, have contributed to human health. Here, a novel hypothesis is proposed, combining the physical principle of MEPP and the biological principle of natural selection. It is hypothesized that nature’s physical efforts serve to augment entropy production, complexity, and function, which complements the evolutionary drive for an additional feature, namely adaptability or capacity to increase the workload (through the activation of fight or flight), measurable by the capacity to augment entropy production if and when required. Both function and adaptability, measured by basal and maximal entropy production, are thus hypothesized to be useful means for measuring health. Basal entropy production is necessary for function, maintenance, and repair (i.e., healing), yet the capacity to augment entropy production and augment the work output would be required for adaptability and the capacity to augment the workload and to evade or respond to illness, heat stress, or other threats, mediated by our autonomic nervous system response. Illness is thus hypothesized to be characterized by a reduction in either resting and/or maximal entropy production, thought to be reflected by a reduction in resting energy expenditure, maximal oxygen consumption, or both; however, if the reduction in maximal consumption is profound, then there will be a compensatory elevation in resting energy expenditure, for example, with COPD(Chronic obstructive pulmonary disease) [71] or sepsis [72]. For example, a patient with severe COPD who is breathing laboriously at rest or a patient with tachycardia secondary to infection may have elevated entropy production at rest, yet their capacity to augment the work output and increase their entropy production will be markedly impaired. While there are many theories for aging [73], it is noteworthy that this approach does not support the “Rate of Living” theory, where a lower basal metabolic rate is associated with longevity, as introduced by Raymond Pearl in 1928, which remains contested [74]. However, VO_2_ max is commonly accepted as the gold-standard measure of cardiopulmonary fitness [75]. As discussed, human entropy production is most readily estimated by oxygen consumption, reflecting metabolism and heat production, assuming a stable temperature. Therefore, overall health, reflecting both function and adaptability, is hypothesized to be related to the ratio (or difference) between resting and maximal entropy production. Although unconfirmed, this is hypothesized to be measurable with the ratio (or difference) between maximal oxygen consumption and resting energy expenditure. This hypothesis may be readily tested with existing data sets. While this exploration on non-equilibrium thermodynamics may already seem too complex, it does not end with metabolic thermodynamic entropy production. As we are cognitive systems, in addition to being complex, non-equilibrium, and adaptive, informational entropy production must also be considered.

## 4. Informational Entropy Production

More than any other living creature, we have developed a remarkably complex central nervous system that performs an additional and vital form of entropy production that appears to have been unexplored. The study of entropy has been performed for a long time in both information science and thermodynamics [76]. Formally linking the two, Landauer’s principle states that any “loss of information” must be accompanied by entropy production to the environment [77], which has been confirmed experimentally [78,79,80,81]. Here, as a philosophical rather than quantitative idea, it is hypothesized that humans not only produce entropy thermodynamically and metabolically, but also through information loss via our consciousness. To clarify, “loss of information” through consciousness is proposed to connote a synthesis of sensory data into meaning, along with storage into memory. Akin to diminishing the resolution of a photograph without losing its meaning, information loss is synonymous with entropy production, and is irreversible; no process can result in a net gain of information over time [82]. Akin to entropy production due to the irreversibilities within metabolism, there are irreversibilities with respect to information management. When one translates an array of information or data into a shorter description indicating meaning or understanding, that synthesis of information occurs with a loss of information, as the meanings or symbols we use to simplify the world never truly reflect all the details of the real image. Language plays a key role in packaging massive external information into bite-sized manageable concepts.

How is this relevant to consciousness? As a thought experiment relevant to every one of us, let us imagine the origins of consciousness in a baby approaching and then experiencing birth. Initial sensory information is vast and largely uncorrelated, yet through repeated experiences, recurring patterns are detected, and meaning ascribed, as consciousness grows. Just considering visible sensory information, imagine the information initially exposed to a newborn infant’s eye and its evolution over time; initially incomprehensible and uncorrelated, this vast array of visual sensory information is slowly reduced into meaning and memory as consciousness forms, remarkably and spontaneously every time, in what subjectively appears to be discrete ‘jumps’ as consciousness evolves during human development. In a spontaneous emergent phenomenon, information is integrated and understood as shapes and symbols (e.g., faces and words) that effectively synthesize and reduce information into manageable quantities. The loss of uncorrelated information and gain of memories, classifications, theories, names, words, etc., is hypothesized to represent human informational entropy production; thus, the emergence and growth of consciousness is thus again hypothesized to be secondary to nature’s drive for maximal entropy production. This information loss and entropy production must occur internally within the brain. This does not only occur with the formation of consciousness; every time we synthesize a complex sensory experience into meaning and memory, we are generating entropy. To summarize, dependent upon, yet distinct from, metabolism, the spontaneous emergence and ongoing functioning of human consciousness require the processing, synthesis, and storage of information, reflecting the ubiquitous phenomenon of nature’s pursuit of entropy production.

## 5. Evaluation and Implications

**Figure 1:**
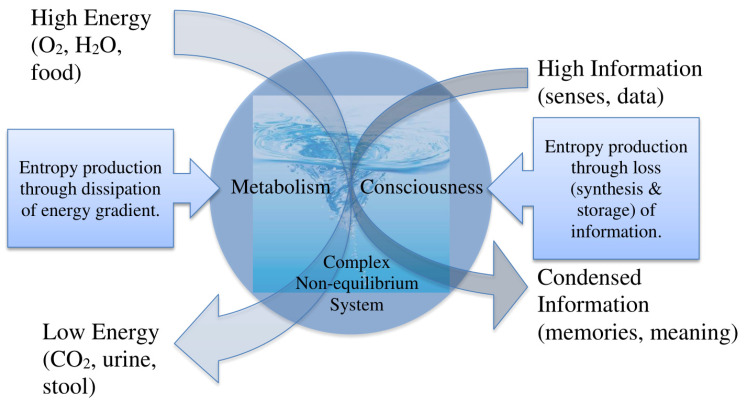
Complex adaptive dissipative cognitive system.

The hypotheses that fractal dissipative structures (e.g. whirlpool) form spontaneously to augment entropy production, that health is characterized by robust human entropy production and the capacity to increase it, and that our entropy production comprises both metabolism and informational processing all merit rigorous evaluation (see Figure 1). Physically, the association between complex fractal structures and flow may be evaluated by evaluating both in broadly disparate systems, analysing whether a growth in flow occurs simultaneously with an increase in complexity (e.g., fractal dimension) of physical models of energy flow (e.g., whirlpools and hurricanes), as well as explored with computational models. Clinically, the hypothesis that health is characterized by both an elevated baseline and capacity to augment entropy production may be tested by measuring the resting and maximal heat production of a wide variety of humans. The following are clinical examples in my clinical experience that support these hypotheses: (1) Illness and frailty are associated with an impaired ability to augment entropy production, work, and/or oxygen consumption if required, and impaired mortality risk from illness; (2) patients with septic shock who do not have the capacity to augment their cardiac output and oxygen delivery (and entropy production) have an increased mortality risk; (3) patients with impaired VO_2_max (<15 mL/kg/min) are high risk for thoracic surgery, and further impairment (<10 mL/kg/min) is considered a prohibitive risk; and (4) intelligence (at least one form of it) appears to be associated with the capacity to synthesize and reduce large masses of information to memorable and communicable summaries. However, the utility of thinking about health and illness in terms of entropy production is really only of interest to clinicians if the ideas offer potential to improve patient care.

### Therapeutic Implications

The overall clinically relevant hypothesis to evaluate is that a targeted focus on optimizing our patient’s entropy production, both at rest and maximally, will improve their health and clinical outcomes. As entropy production is measurable as heat production divided by temperature, and assuming that the temperature remains relatively constant and heat production varies greatly with physical activity (which drives metabolism), entropy production over time is thus closely linked to metabolism over time. While this association merits further investigations to understand its limitations, several investigations support it. Heat production reaches maximal values during near peak exercise (i.e., VO_2_max) maintainable for very short intervals; over longer periods, heat production is predominantly secondary to resting energy expenditure (REE) [83]. Athletes have both elevated resting energy expenditure and the highest levels of maximal oxygen consumption, which can be enhanced with interval exercise training [84]. If oxygen consumption (and entropy production) is markedly reduced (e.g., cardiogenic, hemorrhagic, or distributive shock), efforts to augment it must be instituted to save the patient’s life, by restoring what limiting factor exists (e.g., limitation in the cardiac output, oxygen content, perfusion, or cellular consumption). In patients, ensuring adequate oxygen delivery and ventilation; renal, hepatic, and intestinal waste removal; and early ambulation after critical illness and surgery, all represent foundational therapies that support metabolism and entropy production. We do this already; however, we do not think of it in terms of entropy production. Enhancing entropy production at rest maximally supports interventions such as exercise and heat stress (i.e., sauna) for short periods of time, provided the body is capable of shedding the increased heat required to maintain a normal body temperature. As discussed, entropy production is required for our patients to generate an internal healthy order, which might also be described as healing (a self-organizing process); thus the body’s ability to heal itself may simply reflect nature’s physical drive for maximizing entropy production.

Given that entropy production is heat production divided by temperature, an alteration in temperature offers an additional therapeutic modality if it enhances entropy production. As a response to infection, fever might be viewed as a physiological response, if it augments entropy production, to overcome a threat through stimulation of the host response and metabolism. As fever enhances the metabolic rate and oxygen consumption [78,85,86,87], as well as the immune function [88,89], fever is thus hypothesized to be useful if it augments metabolism and heat production to a greater proportional extent than the rise in temperature, such that entropy production is increased. Therefore, suppressing fever if it is enhancing entropy production in response to infection may be harmful [90,91]. However, in critically ill patients with markedly elevated basal entropy production (i.e., elevated REE), fever is unlikely to further augment metabolism, and thus may not be beneficial; it may even be harmful. If the temperature increases without a greater proportional rise in heat production, overall entropy production is reduced; thus, suppressing fever where entropy production is decreased may be beneficial. Currently, antipyresis used indiscriminately in critically ill patients with sepsis has a minimal effect [92]. Analogously, therapeutic hypothermia, which involves cooling patients, may be useful in helping to maintain or enhance entropy production locally or systemically, provided the cooling does not reduce metabolism and heat production more than the drop in temperature. Certainly, hypothermia is beneficial when energy supplies are deficient, such as after cardiac arrest [93], or for the cryopreservation of tissues and embryos when one wishes to decrease metabolism. In addition, cooling is helpful for avoiding local temperature elevation after joint arthroplasty [94]. As this is a broad avenue for exploration, monitoring entropy production may offer a means to guide when hyperthermia and/or cooling are helpful and when they are not. In summary, temperature alteration is hypothesized to be therapeutic if it augments resting and maximal entropy production.

Given the association of fractal structures with optimal entropy production, monitoring multi-scale self-similar fractal structures may assist with the monitoring of systemic properties helpful for forewarning and/or guiding decision making. Additionally, restoring fractal structures poses a means of augmenting basal and maximal entropy production. Health is associated with fractal fluctuations of the heart and respiratory rate, and illness and aging are associated with the loss of fractal variation [95,96,97,98]. The loss of fractal anatomy (e.g., emphysema and atherosclerosis) impairs the internal organ-specific dissipation of energy gradients. The continuous monitoring of scale-invariant variation to detect when it is altered may offer a means of detecting the early onset of illness, or help to guide decision-making [60,65,99,100]. In addition, restoring scale-invariant life-support offers value in improving oxygen delivery; for example, an improvement in jugular venous oxygen saturation is observed during rewarming from bypass with biologically variable pulsatile (vs. apulsatile or conventional pulsatile) cardiopulmonary bypass [101,102], and biologically variable ventilation improves arterial oxygenation [103,104]. Therefore, monitoring fractal physiology to help make clinical decisions, and/or restoring fractal physiology to directly enhance entropy production, may both prove therapeutic.

Finally, the therapeutic implications of restoring or enhancing information processing and storage as a vital form of informational entropy production merits a brief discussion. Clearly, restoring basal entropy production (i.e., basal consciousness) is a key component of critical care medicine. Avoiding or using light sedation in critically ill patients is part of standard care, as deep sedation is harmful [105,106,107]. A focus on restoring functional consciousness would complement existing guidelines. The question of the potential benefit of enhancing both basal and maximal information processing is worthy of further exploration; are brief periods of intense efforts to utilize information processing and memory therapeutic? Exercising our capacity to process information appears to be beneficial to cognition. We all experience that nature provides a therapeutic benefit; is it related to the depth and complexity of the multi-scale self-similar sensory information (e.g., waves, mountains, trees, clouds, etc.) that our brains must process? Finally, the impact of this approach in promoting psychological health is beyond the scope of this initial discussion, yet merits further study.

## 6. Limitations

These key concepts and testable ideas are offered to enhance discussions and promote investigation (see Table 1). Specifically, the relationship between entropy production, resting energy expenditure, the basal metabolic rate, and the variable role of metabolic efficiency remains to be explored further, both in states of health and illness. The measurement of heat production in health and illness, and the importance of changes in body temperature, merit exploration. The conjecture that information synthesis into consciousness and memories is a source of entropy production merits quantitative modeling and study. With further exploration required, these unproven concepts offer the potential to help bridge physics, biology, and medicine.

## 7. Conclusions

All living systems produce entropy to survive; after a period of growth in entropy production, we lose it slowly during aging or abruptly during illness, and its cessation signifies the end of life. Helping our patients to optimize their entropy production at rest and maximally may assist with health and healing. Physiological and clinical research is required to critically appraise these hypotheses, with the hope that new understanding will lead to improved patient care.

## Figures and Tables

**Table 1:** Key concepts.

**Entropy Production**	While energy is conserved, energy gradients are universally and irreversibly dispersed, producing entropy. Complex non-equilibrium systems that are continuously breaking down energy gradients seek to augment their entropy production (i.e., MEPP). Entropy production equals heat production (Q) divided by temperature (T). Human heat production is largely determined by metabolism, and is greatly impacted by temperature (e.g., fever drives increased metabolism and cooling decreases metabolism). In multiple disparate living structures studied, an initial growth in entropy production is observed, followed by a plateau and then a fall, and its cessation occurs with death.
**Physical Structures**	Fractal structures in nature (i.e., bounded multiscale self-similarity) form spontaneously in order to optimize entropy production. Fractal anatomic (i.e., tree-like) and temporal (e.g., heart rate variability) structures found in human physiology are essential for optimal systemic entropy production.
**Impact of Evolution**	The evolutionary drive for enhanced function and adaptability is hypothesized to select states with both robust basal entropy production and the capacity to augment it when required.
**Informational Entropy Production**	Humans also produce entropy through the synthesis and storage of information into meaning and memory within the central nervous system. The origin of consciousness may reflect nature’s drive to produce entropy.
**Health**	Overall, human health, reflecting both function and adaptability, is hypothesized to be related to elevated resting and maximal entropy production, estimable by the basal resting energy expenditure and maximal oxygen consumption.
**Illness**	Breakdown of fractal structures in space (i.e., vascular networks and tracheobronchial tree) or time (i.e., heart rate variability) occurs with illness. Illness and aging are associated with either a decrease in basal or maximal entropy production, or both.
**Therapeutic Implications**	Optimizing our patient’s entropy production at rest and maximally may improve their health and clinical outcomes. Monitoring the loss of fractal variability to predict clinical outcomes may assist with clinical decision-making. Restoring fractal physiology through biologically variable life support may be useful for enhancing entropy production. Therapeutic temperature alteration may be guided by monitoring the impact on heat production divided by temperature; hyperthermia or cooling may be beneficial if they enhance entropy production.
